# Mapping the Epigenetic Regulatory Landscape and Core Transcriptional Axes Across Cell Cycle Phases in Lung Cancer

**DOI:** 10.3390/ijms27219992

**Published:** 2026-11-07

**Authors:** Isabella Salcedo-Tuiran, César Payán-Gómez, Juan Javier López-Rivera, Luisa Fernanda Patiño-Unibio, Sally Lorena Arboleda-Mojica, Claudia Aristizábal-Guzmán, Carlos Arturo Álvarez-Moreno, Mario Arturo Isaza-Ruget, Beatriz Andrea Otálora-Otálora

**Affiliations:** 1 Fundación Universitaria Sanitas, Bogotá 110131, Colombia; i.salcedotu@unisanitas.edu.co (I.S.-T.); sally.arboleda@unisanitas.edu.co (S.L.A.-M.); claristizabal@unisanitas.edu.co (C.A.-G.); misaza@keralty.com (M.A.I.-R.); 2 Dirección Académica, Universidad Nacional de Colombia, Sede de La Paz, La Paz 111016, Colombia; cepayang@unal.edu.co; 3 Clínica Colsanitas, Laboratorio Clínico y de Patología, Bogotá 111321, Colombia; jjlopez@colsanitas.com; 4 Clínica Colsanitas, Clínica Universitaria Colombia, Bogotá 111321, Colombia; minternacuc@colsanitas.com; 5 Clínica Colsanitas, Departamento de Enfermedades Infecciosas, Bogotá 111321, Colombia; calvarez@colsanitas.com

**Keywords:** lung cancer, epigenetics, cell cycle, non-coding RNAs, microRNAs, chromatin remodeling, CHEK1, regulatory networks

## Abstract

Lung-cancer cell-cycle progression is shaped by interacting checkpoint, transcriptional, chromatin, and non-coding RNA regulatory layers rather than by isolated molecular alterations. This integrative review uses SMARCB1, NOC2L, CDCA4, and CHEK1 as four complementary anchor regulators representing SWI/SNF-dependent chromatin remodeling, inhibition of histone acetyltransferase activity, E2F-linked transcriptional modulation, and checkpoint signaling, respectively. We critically map their literature-supported relationships across G1, S, G2, and M and integrate DNA and histone methylation, acetylation, chromatin remodeling, phosphorylation, ubiquitination, hydroxylation, and non-coding RNA regulation. Importantly, lung-cancer-specific evidence is distinguished from evidence extrapolated from other tumor models, and experimentally established mechanisms are separated from contextual or hypothesis-generating phase assignments. The strongest lung-specific evidence concerns CHEK1 dependence, replication stress, and chromatin-state plasticity, whereas several proposed phase-specific functions of NOC2L, CDCA4, and SMARCB1 remain incompletely validated in lung cancer. Recent 2026 studies further emphasize subtype-specific CHEK1 biology, therapy-associated chromatin reprogramming, and long non-coding RNA control. This framework identifies testable regulatory hypotheses while avoiding interpretation of network association as direct causality.

## 1. Introduction

The mammalian cell cycle is organized into G1, S, G2, and M phases, but the time spent in each phase is not fixed and varies with lineage, proliferative state, nutrient availability, and checkpoint activation. Normal differentiated cells may remain for prolonged periods in G1 or G0 and can delay phase transitions when DNA damage or replication stress is detected. Cancer cells do not simply cycle faster in every context; rather, they commonly increase the cycling fraction, weaken restriction-point control, tolerate chronic replication stress, and rewire checkpoint dependencies. Consequently, phase occupancy becomes heterogeneous across tumor cells and subtypes, with selective pressure favoring cells that can continue proliferating despite genomic and epigenomic stress [1,2]. In lung cancer, these changes occur together with recurrent alterations in DNA methylation, histone modifications, chromatin-remodeling complexes, transcription-factor programs, and non-coding RNA circuits [3,4,5,6].

This distinction between normal and malignant cycling is important because many epigenetic processes are physiologic components of cell-cycle control before they are co-opted by tumors. DNA methylation can preserve transcriptional memory, histone acetylation can tune promoter and enhancer accessibility, ATP-dependent remodeling can reorganize nucleosome occupancy, and microRNAs can dampen transcripts that would otherwise push a cell across a checkpoint [7,8,9,10,11,12]. In cancer, the same regulatory layers can become biased toward proliferation, lineage plasticity, damage tolerance, or therapeutic resistance. Recent work in non-small cell lung cancer (NSCLC), for example, shows that treatment resistance can be accompanied by coordinated chromatin reprogramming and transcriptional-circuit remodeling rather than a single static molecular lesion [13].

The present review is organized around four anchor regulators: SMARCB1, NOC2L, CDCA4, and CHEK1. They were not selected as the four highest-ranked nodes of a new computational analysis. Instead, they were chosen a priori because together they represent complementary regulatory layers that recur in the cell-cycle and epigenetic themes highlighted by the authors’ previously published lung-cancer network frameworks: SMARCB1 represents SWI/SNF-dependent chromatin remodeling; NOC2L represents inhibition of histone acetyltransferase activity and transcriptional repression; CDCA4 represents E2F-linked transcriptional modulation; and CHEK1 represents DNA-damage and replication-stress checkpoint signaling [14,15,16,17,18,19,20]. This anchor-based organization allows the literature to be compared across G1, S, G2, and M without implying that the four proteins constitute a newly discovered physical complex or an unbiased topological ranking.

The objective is therefore not to report a new phase-resolved transcriptomic dataset, but to provide a critical, evidence-aware synthesis of how cell-cycle checkpoints, chromatin regulation, transcriptional control, and non-coding RNAs intersect in lung cancer. Particular attention is given to three distinctions requested during peer review: lung-cancer-specific versus cross-tumor evidence; directly experimentally demonstrated mechanisms versus network- or literature-derived associations; and clinically supported observations versus hypotheses that still require functional validation. The distinctive contribution of this Review is an evidence-graded synthesis that places prior network-derived regulatory context alongside phase-specific mechanistic literature, explicitly separating direct lung-cancer evidence, cross-tumor mechanisms, contextual associations, and testable hypotheses.

## 2. Scope and Evidence-Integration Framework

This manuscript is an integrative narrative review. The previously published transcriptomic and regulatory-network studies from the authors’ group are used as contextual frameworks rather than re-presented as new data [14,15]. The 2024 analysis integrated multiple lung-cancer transcriptomic datasets and reconstructed transcriptional and co-regulatory networks, whereas the subsequent epigenome/exosome study expanded the network context to coding and non-coding RNA-mediated regulation [14,15]. In the present review, these publications motivate the regulatory themes and transcription-factor modules considered, but no de novo network inference, centrality ranking, or phase-resolved transcriptomic analysis is claimed.

A targeted narrative literature search was conducted in PubMed through October 2026 using combinations of “lung cancer”, “NSCLC”, “LUAD”, “LUSC”, “cell cycle”, “epigenetic”, “chromatin”, “non-coding RNA”, “microRNA”, “SMARCB1”, “NOC2L”, “CDCA4”, and “CHEK1”. Primary mechanistic studies and lung-cancer-specific studies were prioritized; recent reviews were used to contextualize broad themes, and publications from 2026 were specifically screened to update the manuscript [2,6,13,21,22,23,24]. When direct lung-cancer evidence was unavailable, evidence from other tumor types or non-malignant models was retained only when it clarified a molecular mechanism and was explicitly identified as cross-tumor evidence. This was a narrative, not systematic, review; no meta-analysis or formal risk-of-bias assessment was performed.

To prevent network association from being interpreted as causality, relationships in the text and figures are classified descriptively. “Mechanistic” relationships refer to experimental evidence supporting a direct regulatory effect or defined molecular interaction. “Lung-cancer-associated” relationships refer to expression, prognosis, functional, or treatment-response data generated in lung-cancer samples or models. “Contextual” relationships integrate prior network context with published biology but do not demonstrate direct causation. “Hypothesis-generating” relationships are used when phase placement or therapeutic interpretation is plausible but lacks direct lung-cancer validation. Supplementary Table S1 maps the principal node-by-phase relationships to these categories and indicates whether the supporting evidence is lung-cancer-specific or extrapolated.

To make the evidence-integration procedure explicit, the four anchors were selected as purposive review-organizing nodes using three criteria: (i) representation of a distinct regulatory layer relevant to cell-cycle control; (ii) recurrence within the themes highlighted by the authors’ prior lung-cancer network studies; and (iii) sufficient mechanistic literature to evaluate phase relevance. This was not an unbiased ranking or an exhaustive node-selection algorithm. No new computational subnetworks were constructed for this Review; the previous subnetwork terminology was removed to avoid implying de novo network extraction. A relationship was treated as phase-specific mechanistic evidence only when a cited primary study directly examined that phase or transition, or when an experimentally established mechanism was explicitly linked to that phase. When phase placement was inferred from broader cell-cycle biology, prior network context, or cross-tumor evidence, it was classified as contextual or hypothesis-generating rather than as phase-resolved evidence. Supplementary Table S1 records the phase/context, summarized relationship, evidence type, lung-cancer specificity, interpretive status, and representative references for the principal relationships. The literature-supported architecture of the four anchor regulators and the distinction between mechanistic and contextual relationships are summarized in Figure 1.

During preparation of the original manuscript, Google Gemini (version information not recorded) was used as an assistive tool to help organize literature-search terms and literature-derived notes. During the major revision and final proofreading, ChatGPT (OpenAI; GPT-5.6 Sol and GPT-6) was the primary AI-assisted tool used to support manuscript restructuring, language editing, organization of the point-by-point revision, organization of evidence classifications and literature verification, and preparation of conceptual figure layouts. An OpenAI image-generation tool (version information not recorded) was used to explore preliminary conceptual figure layouts; the final schematic figures were subsequently redrawn with explicit mechanistic activation and inhibition symbols, using the published literature as the reference for content. None of these tools was treated as a source of scientific evidence; none generated primary experimental data or performed transcriptomic/network inference. Final decisions regarding source inclusion, scientific interpretation, evidence classification, and wording were made by the authors. All cited references, scientific claims, figure content, and interpretations were checked against the cited source material and reviewed by the authors, who retain full responsibility for the content.

## 3. Core Regulatory Nodes: What Is Established and What Remains Contextual

### 3.1. SMARCB1 and SWI/SNF-Dependent Chromatin Control

SMARCB1 (INI1/SNF5) is an obligate subunit of canonical SWI/SNF/BAF chromatin-remodeling complexes and has a well-established tumor-suppressive role in SMARCB1-deficient malignancies [16,25,26]. Mechanistically, SWI/SNF complexes use ATP-dependent remodeling to alter nucleosome positioning and can oppose Polycomb/PRC2-mediated repression. Loss of SMARCB1 can therefore change enhancer accessibility, lineage programs, and the balance between SWI/SNF and EZH2-dependent H3K27 trimethylation [25,27,28,29,30]. These mechanisms are strongly supported across SMARCB1-deficient tumors, but they should not be generalized to common lung cancers without qualification.

Lung-cancer-specific evidence is narrower. SMARCB1 loss has been reported in rare thoracic and pulmonary carcinomas with poorly differentiated or aggressive phenotypes, while alterations of other SWI/SNF components, particularly SMARCA4, are more common in thoracic malignancy [5,31,32,33]. In these rare SMARCB1-deficient entities, loss of INI1/SMARCB1 protein expression defines the deficient state; however, these data do not establish a uniform mRNA-expression or prognostic direction for common LUAD or LUSC. Accordingly, this review does not assign a pan-lung-cancer expression direction to SMARCB1 and treats it as a mechanistically informative chromatin-remodeling anchor rather than a universally altered lung-cancer driver. Its placement across cell-cycle phases reflects the known requirement for chromatin reorganization during transcription, replication, and mitotic exit, but several phase-specific functions proposed in lung cancer remain extrapolations from broader SWI/SNF biology [26,34,35].

### 3.2. NOC2L as an INHAT-Associated Repressor

NOC2L, also known as NIR, is an inhibitor of histone acetyltransferase activity. It can bind histone tails and limit acetylation by coactivators such as p300/CBP, and experimental studies have linked NOC2L to repression of p53-dependent transcription [18,19]. NOC2L has also been described in functional contexts involving AURKB and nuclear repressive complexes, which provides a mechanistic basis for connecting histone-acetylation control with damage-response transcription [19,36]. These observations support NOC2L as a useful anchor for transcriptional repression, but they do not establish a uniform phase-specific role in lung cancer.

Direct lung-cancer evidence for NOC2L remains comparatively limited. In the Human Protein Atlas/TCGA resource cited here, NOC2L is not classified as prognostic in either lung adenocarcinoma (LUAD) or lung squamous cell carcinoma (LUSC) [37]. Accordingly, this review does not assign NOC2L a consistent favorable/adverse prognostic direction or a subtype-specific tumor up-/down-regulation pattern in lung cancer. Its biochemical role as an INHAT-associated repressor is better established than its lung-cancer-specific expression or outcome relevance. Statements that NOC2L suppresses specific G1, S, G2, or M programs in lung cancer are therefore presented as contextual or hypothesis-generating unless a lung-specific primary study supports the claim.

### 3.3. CDCA4 and E2F-Linked Transcriptional Regulation

CDCA4 is an E2F-responsive nuclear factor that modulates E2F-dependent transcription and cell proliferation [17]. This connection provides a direct mechanistic rationale for discussing CDCA4 near the G1/S transcriptional program, where RB-E2F activity governs the induction of DNA-replication genes. However, CDCA4 is not itself a canonical checkpoint kinase, and the evidence does not support describing it as an autonomous sensor that directly determines every phase transition.

Lung-cancer studies indicate context-dependent biology. In NSCLC cell models, Xu et al. reported that CDCA4 overexpression suppressed epithelial–mesenchymal transition, migration, and invasion through an autophagy/CARM1-related mechanism [38]. By contrast, a LUSC study reported high CDCA4 expression in LUSC cells and showed that CDCA4 overexpression promoted proliferation, migration, and invasion and reduced apoptosis; miR-497-5p directly down-regulated CDCA4 and reversed these phenotypes [39]. Importantly, that LUSC study did not identify a significant association of CDCA4 expression with patient survival [39]. Pan-cancer analyses further associate CDCA4 with proliferative phenotypes [40]. These apparently divergent findings justify its inclusion as an E2F-linked transcriptional anchor but argue against a uniform oncogenic or tumor-suppressive direction across lung-cancer contexts. Phase-specific assignments beyond G1/S are therefore interpreted conservatively and mainly through E2F-centered cell-cycle biology [17,41].

### 3.4. CHEK1: The Strongest Lung-Cancer-Specific Checkpoint Anchor

Among the four anchor nodes, CHEK1 has the strongest direct evidence linking cell-cycle checkpoint dependence to lung cancer. ATR-dependent CHEK1 activation coordinates responses to replication stress and DNA damage through targets that include CDC25 phosphatases, thereby restraining CDK activation and delaying inappropriate S-phase progression or mitotic entry [20,42,43]. CHEK1 also contributes to replication-fork stability and can influence chromatin-associated signaling, providing a mechanistic bridge between checkpoint control and the epigenetic context of DNA replication [42,44,45].

Recent 2026 studies refine this picture in a subtype- and context-specific manner. In a TCGA analysis of 492 lung adenocarcinomas and 488 squamous cell carcinomas, high CHEK1 expression in LUAD was associated with advanced T stage, lymph-node metastasis, and shorter overall survival, whereas analogous clinicopathologic and survival associations did not reach statistical significance in LUSC [21]. A separate multi-omic study found CHEK1 expression to be higher in LUAD tumors than in normal-adjacent tissue, linked high CHEK1 expression to worse overall survival, and showed that CHEK1 knockdown reduced proliferation and migration in A549 cells; the same study also connected CHEK1-high tumor cells with an immunosuppressive microenvironment [22]. In addition, C1QBP–STAT1-dependent activation of a c-Myc–CHK1/CHK2 axis was implicated in lung-cancer radioresistance [23]. These observations strengthen the rationale for CHEK1 as a lung-cancer-relevant checkpoint node while also arguing for histology- and treatment-specific interpretation.

The disease-specific evidence, expression/prognostic direction, phase interpretation, and principal limitations for the four anchor regulators are summarized in Table 1.

## 4. Cell-Cycle Phase-Resolved Regulatory Landscape

The phase-based organization below should be interpreted as a literature synthesis rather than as phase-resolved omics data. Exact phase occupancy differs among tumors, and most cited studies do not measure all four anchors simultaneously across synchronized lung-cancer cells. The purpose is to identify where each regulatory layer is most biologically plausible and best supported, while explicitly marking areas in which the phase assignment remains inferential [1,2]. To minimize repetition, the detailed phase-by-node evidence matrix is provided in Supplementary Table S1; the text below highlights only the regulatory features that are most informative for each phase.

### 4.1. G1: Restriction-Point Control and Proliferative Commitment

G1 integrates mitogenic signaling, nutrient status, differentiation state, and DNA-damage surveillance before commitment to DNA synthesis [1]. RB-E2F regulation provides a central transcriptional axis, while chromatin accessibility determines which E2F-responsive and lineage-specific programs can be engaged [10,46,47]. SMARCB1/SWI-SNF activity can oppose inappropriate repressive chromatin states, NOC2L can limit acetylation-dependent transcription, and CDCA4 can modulate E2F-dependent transcription [17,18,19,25]. CHEK1 can influence the G1/S boundary through CDC25A-dependent control when damage signaling is engaged, but its canonical functional dominance is greater after cells enter S phase [20,48]. Therefore, the framework treats G1 as a convergence point rather than assigning equivalent causal weight to all four anchors.

### 4.2. S Phase: Replication, Chromatin Reassembly, and Replicative Stress

S phase provides the strongest rationale for integrating checkpoint and epigenetic regulation. Replication exposes single-stranded DNA and challenges nucleosome organization, requiring coordinated fork protection, histone recycling, and restoration of DNA and histone marks [35,44,49,50,51,52]. ATR-CHEK1 signaling restricts excessive origin firing, stabilizes stressed forks, and restrains CDC25A-CDK2 activity when replication is compromised [42,44,49]. In parallel, CAF-1/CHAF1B and ASF1 family chaperones contribute to nucleosome reassembly, while UHRF1-DNMT1-PCNA coupling supports maintenance DNA methylation behind the fork [50,51,52,53,54,55,56,57,58,59].

The roles of the remaining anchors are less direct. CDCA4 can be placed in S-phase transcription through its E2F relationship, whereas SMARCB1 is relevant to the broader requirement for remodeling and preservation of chromatin architecture [17,26,35]. NOC2L-mediated acetylation blockade is mechanistically compatible with transcriptional restraint, but direct evidence that NOC2L specifically coordinates S-phase progression in lung cancer is limited. For this reason, Figure 2 and Supplementary Table S1 label these relationships by evidence strength rather than presenting them as equivalent established mechanisms.

### 4.3. G2: Premitotic Surveillance and Chromatin Reorganization

G2 is dominated by the decision to enter mitosis only after replication is sufficiently complete and damage has been managed. ATR-CHEK1 signaling can inhibit CDC25B/C and reinforce WEE1-dependent suppression of CDK1, delaying mitotic entry [20,60,61,62,63,64]. At the chromatin level, repair-associated accessibility must be reconciled with the progressive reorganization required for chromosome condensation. SWI/SNF complexes, HDACs, DNA methylation machinery, and histone-modifying enzymes contribute to this transition, although many of these processes are not uniquely G2-specific [10,11,26]. The proposed G2 roles of NOC2L and CDCA4 should therefore be viewed as network-contextual hypotheses unless supported by direct phase-resolved experiments.

### 4.4. M Phase: Checkpoint Signaling and Epigenetic Inheritance

During mitosis, global transcription is reduced, and chromosome architecture is extensively reorganized. Spindle-assembly checkpoint proteins, AURKB, APC/C regulation, and checkpoint kinases coordinate chromosome attachment and anaphase onset [65,66,67,68,69,70,71,72]. CHEK1 has reported mitotic functions, but its best-established cancer role remains the management of replication-associated damage before and during the G2/M transition. Epigenetic inheritance across mitosis depends on persistence or re-establishment of DNA methylation, histone marks, chromatin-bound factors, and nucleosome organization [11,35,73]. SMARCB1 is biologically relevant to chromatin identity, whereas the placement of NOC2L and CDCA4 in a dedicated lung-cancer mitotic program remains largely hypothesis-generating. These relationships should not be interpreted as proven phase-specific causal mechanisms.

## 5. Epigenetic and Non-Coding RNA Regulatory Layers

### 5.1. DNA and Histone Methylation

DNA methylation is a major mechanism of transcriptional memory in lung cancer. Promoter hypermethylation can silence tumor-suppressive programs, while broader methylation changes vary by histological subtype, exposure history, and genomic context [4,74]. During replication, UHRF1 recognizes hemimethylated DNA and recruits DNMT1, with PCNA and chromatin-assembly machinery helping to restore methylation patterns on daughter strands [53,54,55,56,57,58,59]. Histone methylation adds a second layer: EZH2-mediated H3K27me3 can stabilize repressive chromatin, whereas the balance between PRC2 and SWI/SNF activity influences lineage and proliferation programs [25,27,28,29,30,57]. These mechanisms are relevant across the cycle, and methylation should not be described as an exclusively phase-specific mechanism.

Hydroxylation and active DNA demethylation further complicate this landscape. TET enzymes oxidize 5-methylcytosine and depend on alpha-ketoglutarate, linking epigenetic state to metabolism; the oncometabolite 2-hydroxyglutarate can inhibit alpha-ketoglutarate-dependent dioxygenases [75,76]. Loss of 5-hydroxymethylcytosine and altered TET expression have been reported in lung tumors [77,78,79,80]. These observations support a role for dynamic methylation states in tumor biology, but direct attribution of individual hydroxylation events to specific cell-cycle phases remains limited.

### 5.2. Acetylation, Deacetylation, and Chromatin Remodeling

Histone and non-histone acetylation can rapidly alter protein function and chromatin accessibility. p300/CBP and other lysine acetyltransferases promote acetylation-dependent transcription in selected contexts, whereas HDACs remove acetyl groups and participate in both transcriptional repression and DNA-damage responses [10,11,81,82]. NOC2L adds a distinct mechanism by inhibiting histone acetyltransferase access rather than functioning as a deacetylase [18,19]. The miR-449a-HDAC1 relationship provides a lung-cancer-relevant example of post-transcriptional control over this layer [83,84].

Chromatin remodeling is equally context-dependent. SWI/SNF complexes can facilitate or restrict accessibility depending on genomic location and interacting factors, and alterations of SWI/SNF genes are recurrent in lung cancer [5,26,85]. Importantly, a 2026 NSCLC study demonstrated that osimertinib resistance can involve a coordinated transcriptional circuit coupled to enhancer and chromatin reprogramming [13]. This recent evidence supports the view that therapeutic adaptation can involve network-level reconfiguration of chromatin and transcription, rather than only static mutation-based mechanisms.

### 5.3. Phosphorylation and Ubiquitin-Dependent Cell-Cycle Control

Phosphorylation and ubiquitination are not epigenetic modifications in the narrow sense, but they determine the abundance and activity of proteins that write, erase, read, or respond to chromatin states. CHEK1-mediated phosphorylation of CDC25 proteins illustrates how checkpoint signaling is translated into altered CDK activity [20,42,60,61,62,63,64]. Ubiquitin ligases such as SCF complexes and APC/C regulate turnover of cyclins, phosphatases, and other transition factors, providing temporal directionality to the cell cycle [86,87,88,89,90,91,92,93,94,95]. These post-translational systems are therefore included as regulatory interfaces rather than labeled as classical epigenetic mechanisms.

### 5.4. Non-Coding RNAs as Integrators of Cell-Cycle and Epigenetic Programs

Non-coding RNAs are particularly relevant to this Special Issue because they connect checkpoint, transcriptional, and chromatin pathways at the post-transcriptional level. Primary lung-cancer studies support several specific axes. In lung-cancer models, miR-449a/b directly bound the HDAC1 3′ untranslated region; miR-449a/b was down-regulated, HDAC1 was up-regulated, and combining miR-449a with HDAC inhibition produced greater growth arrest than HDAC inhibition alone [84]. Earlier direct targeting of HDAC1 by miR-449a was demonstrated in prostate cancer and is cited here as cross-tumor mechanistic context [83]. In lung-cancer cells, miR-101 reduced EZH2 expression and invasion-related phenotypes [96], while broader EZH2–miRNA cross-talk has been reviewed separately [97]. In LUSC, miR-497-5p directly down-regulated CDCA4 and restrained proliferative, migratory, and invasive phenotypes [39]. In NSCLC, miR-195 directly targeted CHEK1, was reduced in tumor tissue, and lower miR-195 together with higher CHEK1 was associated with worse survival in the reported cohort [98]. For broader mechanistic context, reviews of miRNAs in DNA-damage and cell-cycle control [99,100,101] and primary cross-tumor studies of miR-182–BRCA1 and miR-34a–E2F [102,103] are treated as contextual rather than lung-cancer-specific evidence.

The non-coding RNA landscape also extends beyond microRNAs. A 2026 study identified the glucose/glutamine-sensitive lncRNA GSLR as a regulator of lung adenocarcinoma progression, linking chromatin accessibility, CTCF-dependent transcription, RNA-helicase recruitment, and metabolic homeostasis [24]. This example illustrates why non-coding RNA regulation should not be treated as a peripheral add-on to epigenetics: RNA species can participate in feedback circuits that influence chromatin, transcription, metabolism, and cell-state plasticity. At the same time, the mechanistic specificity of individual ncRNAs means that broad claims about a universal “ncRNA cell-cycle program” should be avoided.

The major regulatory layers, representative components, lung-cancer-specific evidence, and interpretive cautions discussed in this Review are summarized in Table 2.

## 6. Critical Synthesis: Lung-Cancer Specificity, Causality, and Evidence Gaps

A central conclusion of this review is that the evidence is uneven across the four anchor regulators. CHEK1 has the clearest lung-cancer-specific support, including expression/progression data, functional studies, and recent evidence relating checkpoint signaling to radioresistance and tumor-microenvironment biology [21,22,23,43]. CDCA4 has direct lung-cancer studies, but its direction of effect is context-dependent: it can participate in proliferative E2F programs while individual functional studies also report effects on migration, invasion, autophagy, or microRNA-mediated repression [38,39]. SMARCB1 has strong mechanistic support as a tumor suppressor and chromatin-remodeling component, but direct evidence in common lung-cancer subtypes is limited because complete SMARCB1 loss is rare [5,31,32,33]. NOC2L has a defined biochemical mechanism but comparatively sparse lung-cancer-specific functional validation [18,19,37].

These differences also affect how network relationships should be interpreted. Co-expression, shared pathway annotation, or placement in a regulatory module does not prove direct binding or causal regulation. Likewise, the fact that two proteins are relevant to the same phase does not prove that they act in a single pathway. This review therefore uses “associated with”, “contextually linked to”, or “consistent with” when the evidence is indirect, and reserves terms such as “phosphorylates”, “binds”, “represses”, or “activates” for mechanisms supported by cited experiments. These distinctions are reflected in the text, Figure 1 and Figure 2, and Supplementary Table S1.

Histological subtype is another major source of heterogeneity. LUAD, LUSC, and SCLC have distinct genomic and epigenomic landscapes, and recent CHEK1 data illustrate that an association observed in LUAD may not reproduce in LUSC [21]. Therefore, pan-lung-cancer claims should be interpreted cautiously unless the supporting study includes multiple subtypes. The review now identifies subtype-specific findings when they are available and explicitly labels evidence from other tumor models rather than treating it as interchangeable with lung-cancer evidence.

Finally, the phase-resolved framework is best viewed as a hypothesis-generating map. Most public lung-cancer transcriptomic datasets are not synchronized or annotated by cell-cycle phase, and the prior network frameworks were not designed as phase-resolved datasets [14,15]. Direct validation will require synchronized or single-cell experimental systems, perturbation of individual anchors, phase-aware chromatin profiling, and orthogonal assays of physical interaction and causality. These limitations do not negate the value of the framework; instead, they define which relationships are sufficiently established to guide interpretation and which should become the focus of future experiments.

## 7. Therapeutic Implications and Future Directions

The translational relevance of this framework lies primarily in identifying testable candidate dependencies rather than validated biomarkers or drug combinations. Epigenetic therapies in NSCLC have historically included DNA methyltransferase- and HDAC-directed strategies, while newer work continues to examine EZH2, chromatin remodeling, and other state-regulating mechanisms [6,104,105]. However, response is strongly influenced by histology, driver genotype, prior therapy, and the specific epigenetic state of the tumor. Importantly, this review does not perform diagnostic or prognostic validation and therefore does not designate any molecule as a validated biomarker; external cohort associations are discussed as literature evidence only. Likewise, the review does not propose any drug combination as clinically established solely on the basis of the integrated framework.

CHEK1 provides the clearest example of a preclinically supported candidate dependency that still requires context-specific validation. Recent studies associate CHEK1 with LUAD progression and an immunosuppressive tumor state, while experimental work links CHEK1-related signaling to radioresistance and sensitivity to CHEK1-directed perturbation in lung-cancer models [21,22,23,106]. These data justify further investigation of checkpoint inhibition, particularly in tumors with high replication stress or defective parallel damage-response pathways, but they do not establish a universal biomarker or standard combination regimen. Similarly, a 2026 study of osimertinib-resistant NSCLC identified a resistance-specific transcriptional/chromatin circuit with experimentally tractable downstream dependencies [13]; that observation is presented as model-specific evidence rather than a general therapeutic rule for NSCLC.

For non-coding RNAs, therapeutic translation remains preclinical. miRNA restoration or inhibition could in principle alter HDAC, EZH2, CDCA4, CHEK1, and other cell-cycle-related programs, but delivery, target specificity, tumor heterogeneity, durability, and off-target effects remain substantial barriers. The emerging lncRNA literature, including GSLR in LUAD, expands the set of experimentally testable regulatory targets but likewise requires independent validation before biomarker or therapeutic claims are warranted [24]. A productive next step would be to combine phase-aware single-cell transcriptomics with chromatin accessibility and perturbation screens to determine which regulatory dependencies persist within clinically relevant lung-cancer subtypes and treatment states.

## 8. Conclusions

Lung-cancer cell-cycle regulation emerges from the interaction of checkpoint signaling, chromatin organization, transcriptional control, and non-coding RNA-mediated feedback. Using SMARCB1, NOC2L, CDCA4, and CHEK1 as complementary anchor regulators helps organize this complexity, but the evidence supporting each node is not equivalent. CHEK1 has the strongest direct lung-cancer-specific checkpoint evidence; CDCA4 has context-dependent lung-cancer associations; SMARCB1 is mechanistically well established but most directly relevant to rare deficient tumors; and NOC2L remains comparatively under-validated in lung cancer. The phase-resolved model presented here should therefore be interpreted as an evidence-graded conceptual framework rather than proof of phase-specific causality. By explicitly separating direct mechanisms, lung-cancer associations, cross-tumor evidence, and hypotheses, this review defines a more rigorous basis for future experiments and for evaluating potential therapeutic vulnerabilities without overstating biomarker or treatment readiness.

## Figures and Tables

**Figure 1:**
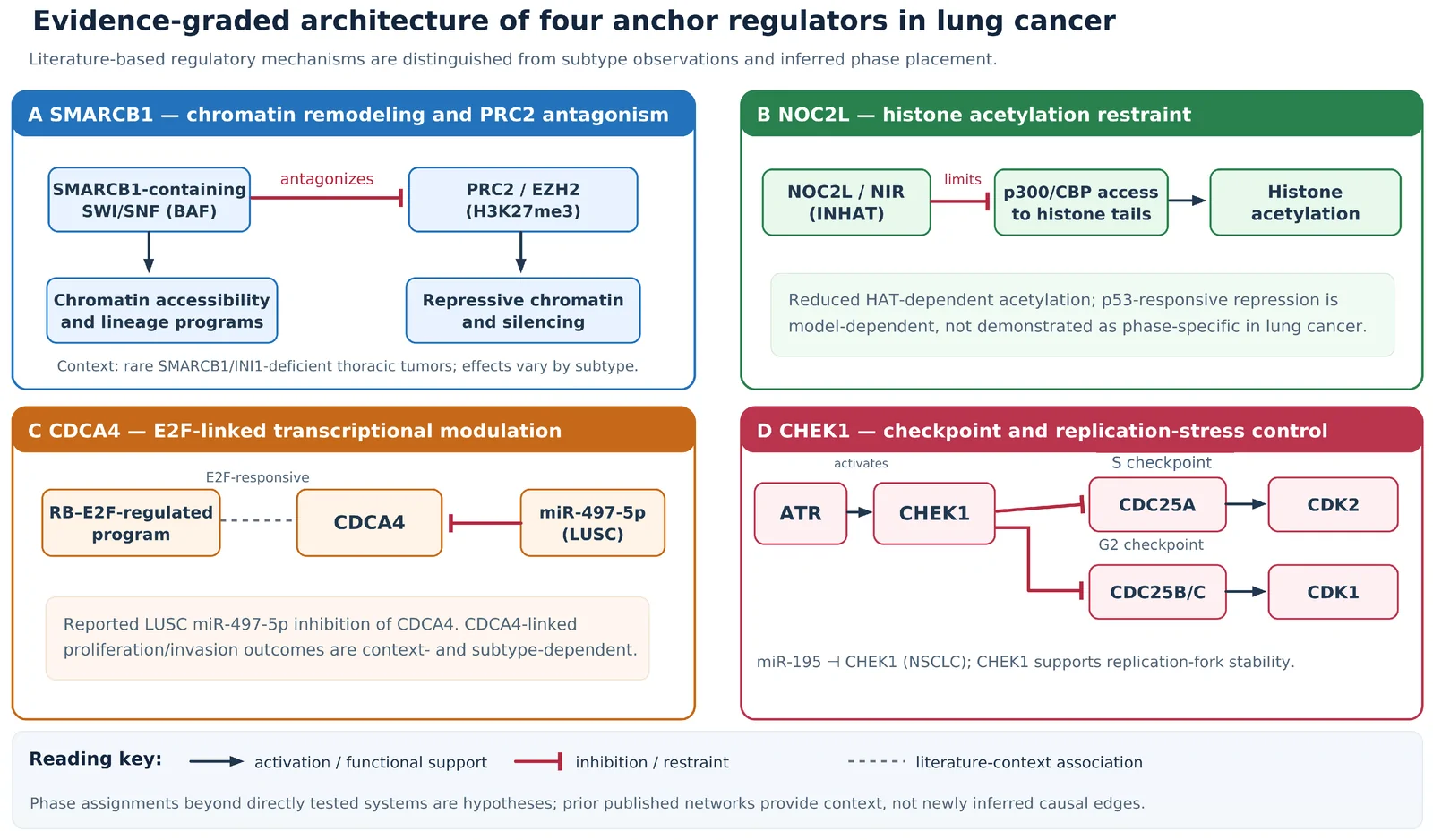
Evidence-graded regulatory architecture of SMARCB1, NOC2L, CDCA4, and CHEK1 in lung-cancer cell-cycle control. Panels (**A**–**D**) summarize literature-supported functions without implying a newly inferred four-node complex. Arrowheads denote activation or functional support, T-bars denote inhibition or restraint, and gray dashed lines denote contextual associations rather than experimentally established direct interactions. SMARCB1-containing SWI/SNF functionally antagonizes PRC2/EZH2; NOC2L reduces histone acetyltransferase access; CDCA4 is linked with E2F-responsive transcription and miR-497-5p-mediated repression in LUSC; and ATR-activated CHEK1 restrains CDC25 family phosphatases and downstream CDK activation. Evidence derived from lung cancer is distinguished from mechanisms established in other systems; phase assignments beyond directly tested models remain hypothesis-generating. Prior network publications provide regulatory context; no de novo subnetwork inference or causal ranking is claimed. Abbreviations: SMARCB1, SWI/SNF-related matrix-associated actin-dependent regulator of chromatin subfamily B member 1; SWI/SNF, switch/sucrose non-fermentable; BAF, BRG1/BRM-associated factor; PRC2, Polycomb repressive complex 2; EZH2, enhancer of zeste homolog 2; H3K27me3, histone H3 lysine 27 trimethylation; NOC2L, nucleolar protein 2-like; NIR, alternative name for NOC2L; CDCA4, cell division cycle associated 4; CHEK1, checkpoint kinase 1; p300, histone acetyltransferase p300; p53, tumor protein p53; INHAT, inhibitor of histone acetyltransferase; HAT, histone acetyltransferase; CBP, CREB-binding protein; RB, retinoblastoma protein; E2F, E2F transcription factor family; ATR, ataxia telangiectasia and Rad3-related kinase; CDC25, cell division cycle 25 phosphatase; CDK, cyclin-dependent kinase; LUSC, lung squamous cell carcinoma; NSCLC, non-small-cell lung cancer; miR, microRNA.

**Figure 2:**
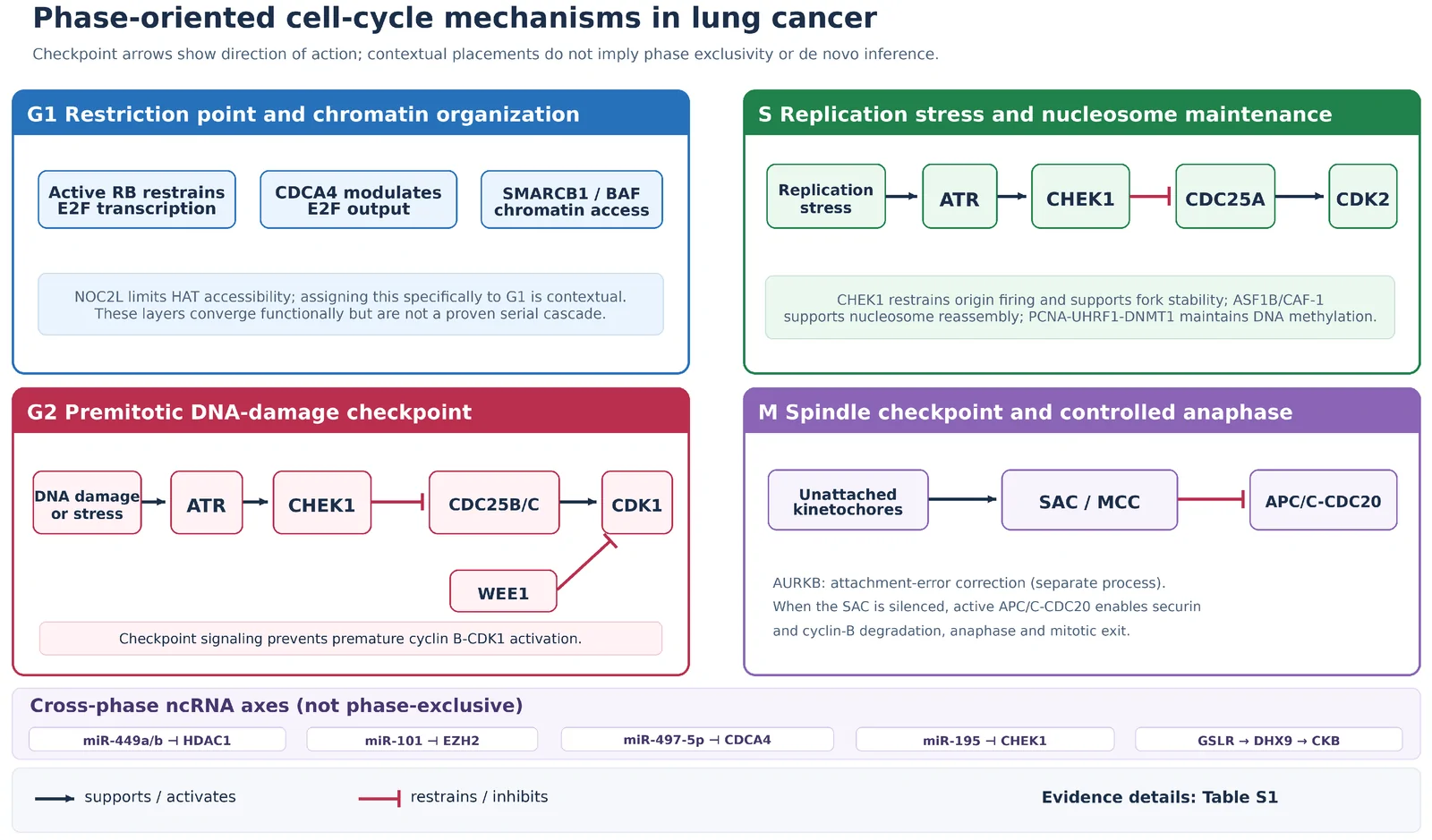
Phase-oriented epigenetic and checkpoint mechanisms across the lung-cancer cell cycle. G1 summarizes restriction-point regulation and chromatin accessibility without implying a proven linear signaling cascade. S phase illustrates replication-stress-dependent ATR–CHEK1 signaling, CDC25A restraint, replication-fork protection, nucleosome reassembly, and maintenance DNA methylation. G2 phase illustrates CHEK1-dependent inhibition of CDC25B/C and suppression of premature CDK1 activation, together with inhibitory WEE1 signaling. During M phase, the spindle-assembly checkpoint and mitotic checkpoint complex (SAC/MCC) restrain APC/C–CDC20 until proper kinetochore attachment; Aurora B kinase (AURKB) contributes to attachment-error correction rather than directly activating APC/C. Arrowheads indicate activation or functional support, T-bars indicate inhibition. The ncRNA axes in the lower band are not assigned exclusively to individual phases. The diagram synthesizes published findings; it is not new network inference or evidence that every phase assignment is lung-cancer-specific. Abbreviations not defined in Figure 1: ASF1B, anti-silencing function 1B histone chaperone; CAF-1, chromatin assembly factor 1; PCNA, proliferating cell nuclear antigen; UHRF1, ubiquitin-like with PHD and RING finger domains 1; DNMT1, DNA methyltransferase 1; WEE1, WEE1 G2 checkpoint kinase; SAC, spindle assembly checkpoint; MCC, mitotic checkpoint complex; APC/C, anaphase-promoting complex/cyclosome; CDC20, cell division cycle 20; AURKB, Aurora kinase B; ncRNA, non-coding RNA; HDAC1, histone deacetylase 1; GSLR, glucose/glutamine-sensitive long non-coding RNA; DHX9, DExH-box helicase 9; CKB, creatine kinase B.

**Table 1:** Evidence-aware summary of the four anchor regulators. The table reports direction-specific lung-cancer expression/prognostic evidence when available and distinguishes these observations from phase interpretation; it is not a quantitative ranking.

Anchor Regulator	Principal Regulatory Layer	Lung-Cancer Expression/Prognostic Evidence	Phase Interpretation and Main Limitation
SMARCB1	SWI/SNF chromatin remodeling; PRC2/EZH2 antagonism	Rare SMARCB1/INI1-deficient thoracic/lung tumors show protein loss; no uniform common-LUAD/LUSC expression or prognostic direction is assigned [5,31,32,33]	Chromatin-remodeling anchor; phase roles are partly extrapolated, and evidence is limited in common lung-cancer subtypes.
NOC2L	INHAT-mediated acetylation blockade; p53 transcriptional repression	HPA/TCGA: NOC2L is not prognostic in LUAD or LUSC [37]	Lung-specific functional data are sparse; G1/S/G2/M assignments are mainly contextual or hypothesis-generating.
CDCA4	E2F-linked transcriptional modulation	LUSC cells: high CDCA4 with pro-growth/pro-invasive phenotype but no survival association [39]; an NSCLC study showed suppressive EMT/migration effects [38]	Strongest rationale near G1/S; functional direction is context dependent and later-phase assignments are inferential.
CHEK1	ATR-checkpoint signaling and replication-stress tolerance	LUAD: high CHEK1 associated with advanced T stage, nodal metastasis, and shorter OS [21]; higher tumor expression/worse OS plus functional dependence [22]	Strongest evidence in S/G2; additional roles and therapeutic dependence vary by subtype and treatment context.

Note: HPA, Human Protein Atlas; TCGA, The Cancer Genome Atlas; LUAD, lung adenocarcinoma; LUSC, lung squamous cell carcinoma; NSCLC, non-small-cell lung cancer; OS, overall survival; EMT, epithelial–mesenchymal transition.

**Table 2:** Major regulatory layers discussed in the review and the strength of their lung-cancer-specific interpretation.

Regulatory Layer	Representative Components	Cell-Cycle and Lung-Cancer Evidence	Interpretive Caution
DNA/histone methylation	DNMT1, UHRF1, EZH2, TET enzymes	Maintenance methylation during replication; promoter/subtype-associated methylation and EZH2/TET alterations in lung cancer [4,57,77,78,79,80]	Effects are context-dependent; phase specificity is often inferred
Acetylation/deacetylation	p300/CBP, HDAC1/2, NOC2L	Rapid control of transcription and repair accessibility; lung-cancer evidence includes HDAC–microRNA relationships and broader NSCLC epigenetic dysregulation [6,83,84,104]	Direct NOC2L functional evidence in lung cancer remains limited
Chromatin remodeling	SWI/SNF/SMARCB1, ACTL6A, CHAF1B	Nucleosome accessibility and replication-associated reassembly; SWI/SNF alterations and treatment-associated chromatin reprogramming occur in lung cancer [5,13]	SMARCB1-specific evidence is strongest in rare deficient tumors
Checkpoint phosphorylation	ATR-CHEK1, WEE1, CDC25, CDKs	Replication stress and G2/M control; strong LUAD/checkpoint and radioresistance evidence [21,22,23]	Dependency varies by histological subtype and genetic context
Ubiquitination	SCF ligases, APC/C, FBXW7	Ordered turnover of cell-cycle regulators; lung-cancer relevance depends on the specific target and context [86,87,88,89,90,91,92,93,94,95]	Regulatory interface rather than a classical epigenetic mark
Non-coding RNAs	miR-449a, miR-101, miR-497-5p, GSLR	Post-transcriptional feedback with epigenetic/checkpoint genes; direct lung-cancer examples are available [24,39,84,96]	Targets and direction of effect are highly context-specific

Note: NSCLC, non-small-cell lung cancer; LUAD, lung adenocarcinoma; HDAC, histone deacetylase; APC/C, anaphase-promoting complex/cyclosome; miR, microRNA.

## Data Availability

No new primary datasets were generated or analyzed for this review. The previously published transcriptomic and regulatory-network frameworks discussed here are available in the original publications and their associated repositories/Supplementary Files [14,15]. Supplementary Table S1 provides the evidence classification used in the revised synthesis.

## Supplementary Materials

The following supporting information can be downloaded at: https://www.mdpi.com/article/10.3390/ijms27219992/s1.
