# Supplementary material for: Mapping the Epigenetic Regulatory Landscape and Core Transcriptional Axes Across Cell Cycle Phases in Lung Cancer
Source: Int J Mol Sci. 2026 Nov 7;27(21):9992. doi: 10.3390/ijms27219992 (PMC13763743; doi:10.3390/ijms27219992)
Supplement: Supplementary file 1 [file ijms-27-09992-s001.zip › ijms-4626670-supplementary.pdf]

Supplementary Table S1. Evidence map for phase-by-node relationships

The table maps the principal regulatory nodes and non-coding RNA axes across cell-cycle phases or functional contexts. “Mechanistic” refers to experimentally supported molecular effects, which do not necessarily establish a phase-specific effect in lung cancer; “lung-cancer-associated” denotes findings in lung-cancer samples or models; “contextual” and “hypothesis-generating” identify relationships or phase assignments not directly established in lung cancer. This is a qualitative narrative evidence map, not a quantitative strength score or a formal risk-of-bias assessment. Representative references correspond to the main manuscript; they do not necessarily establish all proposed phase assignments.

Node/axis	Phase/context	Relationship summarized	Evidence type	Lung-cancer specificity	Interpretive status	Representative references
**SMARCB1**	G1	SWI/SNF-mediated chromatin accessibility; functional antagonism of PRC2/EZH2	Mechanistic (broader tumors); lung association	Cross-tumor mechanisms; rare SMARCB1-deficient thoracic/lung tumors	Established chromatin mechanism; G1 role in common lung cancer remains contextual	[16,25,27–33]
**SMARCB1**	S	Chromatin remodeling and replication-associated nucleosome organization	Contextual	Primarily cross-tumor	Hypothesis-generating phase placement in lung cancer	[26,34,35]
**SMARCB1**	G2	Premitotic chromatin reorganization	Contextual	Primarily cross-tumor	Hypothesis-generating phase placement in lung cancer	[25,26]
**SMARCB1**	M	Potential involvement in re-establishing chromatin identity after mitosis	Contextual	Broader SWI/SNF biology; rare lung-specific deficient tumors	M-phase role of SMARCB1 not established by cited lung-cancer studies	[5,26,31–33]
**NOC2L**	G1	INHAT-associated restriction of acetylation; p53-linked transcriptional repression	Mechanistic (general)	Mechanism mainly established outside lung cancer	Established repression mechanism; lung G1 specificity not demonstrated	[18,19,36]
**NOC2L**	S	INHAT-mediated transcriptional repression as a possible S-phase context	Contextual; no direct S-phase evidence	General repression mechanism; no lung S-phase validation	Hypothesis-generating; not demonstrated in this phase	[18,19,37]
**NOC2L**	G2	Potential transcriptional repression during premitotic regulation	Contextual; no direct G2 evidence	General NOC2L mechanism; no lung G2-phase validation	Hypothesis-generating; not demonstrated in this phase	[19]
**NOC2L**	M	No defined mitotic mechanism; included to show an evidence gap	Contextual; no direct M-phase evidence	No identified lung-specific mitotic validation	No established M-phase-specific function	[18,19]
**CDCA4**	G1 (G1/S transition)	E2F-responsive transcriptional modulation	Mechanistic (general); lung-cancer-associated	E2F link established; context-dependent NSCLC/LUSC findings	G1/S placement is inferential in lung cancer; effects differ by model	[17,38,39]
**CDCA4**	S	E2F-linked replication-gene program (proposed)	Contextual	Lung association; phase assignment inferred	Contextual, not directly phase-resolved in lung cancer	[17,38,40]
**CDCA4**	G2	Potential link with E2F-regulated G2/M transcription	Contextual	Limited phase-resolved lung-cancer data	Hypothesis-generating phase placement	[17,40,41]
**CDCA4**	M	Possible postmitotic transcriptional re-entry in an E2F-related context	Contextual	No direct M-phase lung-cancer validation	Hypothesis-generating; not demonstrated for CDCA4 in mitosis	[17,41]
**CHEK1**	G1 (G1/S transition)	Contextual connection to CDC25A/CDK2 regulation; CHK1-dependent CDC25A degradation is demonstrated mainly in S/G2	Contextual for G1; mechanistic in S/G2	General mechanism includes lung-derived H1299 cell evidence, but not G1-specific evidence	G1/S placement remains inferential, not direct G1-phase validation	[20,43,48]
**CHEK1**	S	Replication-fork stability, replication-stress signaling, and CDC25A down-regulation	Mechanistic; lung-cancer-associated	General fork evidence; S-phase checkpoint shown in H1299 lung-cancer cells [48]	S-phase function supported experimentally; generalization across lung subtypes limited	[21–23,42–44,48,49]

Node/axis	Phase/context	Relationship summarized	Evidence type	Lung-cancer specificity	Interpretive status	Representative references
**CHEK1**	G2	CDC25A/B/C restraint and parallel WEE1-dependent CDK1 inhibition	Mechanistic; lung-cancer-associated	G2 checkpoint evidence includes H1299 lung-cancer cells [48]; broader mechanisms general	G2 checkpoint well supported; subtype-specific generalization remains limited	[20,21,48,60–64]
**CHEK1**	M	Mitotic and spindle-checkpoint contribution	Mechanistic (general); contextual	Mixed; limited lung-specific mitotic data	Experimentally described mitotic roles; lung phase interpretation limited	[65–72]
**miR-449a/b–HDAC1**	Multiple	Post-transcriptional repression of HDAC1 and growth arrest	Mechanistic; lung-cancer-specific	Direct lung-cancer cell-model evidence	Supported molecular axis; not exclusively phase-specific	[83,84]
**miR-101–EZH2**	Multiple	Post-transcriptional reduction of EZH2	Mechanistic; lung-cancer-specific	Lung-cancer cell-model evidence; broader review context	Supported molecular axis; not exclusively phase-specific	[96,97]
**miR-497-5p–CDCA4**	G1/S context	Direct repression of CDCA4	Mechanistic; LUSC-specific	Direct LUSC model evidence	Supported molecular axis; G1/S placement is contextual	[39]
**miR-195–CHEK1**	Multiple	Direct post-transcriptional repression of CHEK1	Mechanistic; NSCLC-specific	Direct NSCLC cell and cohort evidence	Supported molecular axis; not exclusively phase-specific	[98]
**GSLR–DHX9–CKB**	Cell state/metabolic	CTCF-regulated GSLR recruits DHX9 to the CKB promoter, supporting CKB expression and energy homeostasis	Mechanistic; LUAD-specific	Direct LUAD model evidence (2026)	Supported reported axis; no exclusive cell-cycle-phase assignment	[24]

Abbreviations: ATR, ataxia telangiectasia and Rad3-related kinase; CDK, cyclin-dependent kinase; CDC25, cell division cycle 25 phosphatase; CHEK1/CHK1, checkpoint kinase 1; CKB, creatine kinase B; CTCF, CCCTC-binding factor; DHX9, DExH-box helicase 9; E2F, E2F transcription-factor family; EZH2, enhancer of zeste homolog 2; GSLR, glucose/glutamine-sensitive long non-coding RNA; HDAC1, histone deacetylase 1; INHAT, inhibitor of histone acetyltransferases; lncRNA, long non-coding RNA; LUAD, lung adenocarcinoma; LUSC, lung squamous cell carcinoma; miR, microRNA; NSCLC, non-small-cell lung cancer; PRC2, Polycomb repressive complex 2; SWI/SNF, switch/sucrose non-fermentable.
